# Family Relationship, Water Contact and Occurrence of Buruli Ulcer in Benin

**DOI:** 10.1371/journal.pntd.0000746

**Published:** 2010-07-13

**Authors:** Ghislain Emmanuel Sopoh, Yves Thierry Barogui, Roch Christian Johnson, Ange Dodji Dossou, Michel Makoutodé, Sévérin Y. Anagonou, Luc Kestens, Françoise Portaels

**Affiliations:** 1 Centre de Dépistage et de Traitement de l'Ulcère de Buruli (CDTUB) d'Allada, Allada, Bénin; 2 Centre de Dépistage et de Traitement de l'Ulcère de Buruli (CDTUB) de Lalo, Lalo, Bénin; 3 Programme National de Lutte contre la Lèpre et l'Ulcère de Buruli (PNLLUB), Cotonou, Bénin; 4 Institut Régional de Santé Publique (IRSP), Ouidah, Bénin; 5 Laboratoire de Référence des Mycobactéries (LRM), PNT, Cotonou, Bénin; 6 Immunology Unit, Department of Microbiology, Institute of Tropical Medicine, Antwerpen, Belgium; 7 Mycobacteriology Unit, Department of Microbiology, Institute of Tropical Medicine, Antwerpen, Belgium; Kwame Nkrumah University of Science and Technology (KNUST) School of Medical Sciences, Ghana

## Abstract

**Background:**

*Mycobacterium ulcerans* disease (Buruli ulcer) is the most widespread mycobacterial disease in the world after leprosy and tuberculosis. How *M. ulcerans* is introduced into the skin of humans remains unclear, but it appears that individuals living in the same environment may have different susceptibilities.

**Objectives:**

This study aims to determine whether frequent contacts with natural water sources, family relationship or the practice of consanguineous marriages are associated with the occurrence of Buruli ulcer (BU).

**Design:**

Case control study.

**Setting:**

Department of Atlantique, Benin.

**Subjects:**

BU-confirmed cases that were diagnosed and followed up at the BU detection and treatment center (CDTUB) of Allada (Department of the Atlantique, Benin) during the period from January 1st, 2006, to June 30th, 2008, with three matched controls (persons who had no signs or symptoms of active or inactive BU) for age, gender and village of residence per case.

**Main Outcomes Measured:**

Contact with natural water sources, BU history in the family and the practice of consanguineous marriages.

**Results:**

A total of 416 participants were included in this study, including 104 cases and 312 controls. BU history in the family (p<0.001), adjusted by daily contact with a natural water source (p = 0.007), was significantly associated with higher odds of having BU (OR; 95% CI = 5.5; 3.0–10.0). The practice of consanguineous marriage was not associated with the occurrence of BU (p = 0.40). Mendelian disorders could explain this finding, which may influence individual susceptibility by impairing immunity.

**Conclusion:**

This study suggests that a combination of genetic factors and behavioral risk factors may increase the susceptibility for developing BU.

## Author Summary

*Mycobacterium ulcerans* disease (Buruli ulcer) is the most widespread mycobacterial disease in the world after leprosy and tuberculosis. How *M. ulcerans* is introduced into the skin of humans remains unclear, but it appears that individuals living in the same environment may have different susceptibilities. This case control study aims to determine whether frequent contacts with natural water sources, family relationship or the practice of consanguineous marriages are associated with the occurrence of Buruli ulcer (BU). The study involved 416 participants, of which 104 BU-confirmed cases and 312 age, gender and village of residence matched controls (persons who had no signs or symptoms of active or inactive BU). The results confirmed that contact with natural water sources is a risk factor. Furthermore, it suggests that a combination of genetic factors may constitute risk factors for the development of BU, possibly by influencing the type of immune response in the individual, and, consequently, the development of BU infection per se and its different clinical forms. These findings may be of major therapeutic interest.

## Introduction

*Mycobacterium ulcerans* disease, commonly named Buruli Ulcer (BU), is the most common mycobacterial disease in the world after leprosy and tuberculosis. This emerging disease has been reported in more than 30 countries in Africa, Latin America, Oceania and Asia [1] causing immense human suffering [2], [3], while its prevalence in most endemic countries is uncertain [4]. How exactly *M. ulcerans* is introduced into the skin of humans remains unknown, but in contrast to tuberculosis (TB) or leprosy, the infection is acquired directly or indirectly from the environment and not through contact with other patients [5]. There is now evidence that *M. ulcerans* is an environmental pathogen transmitted to humans from aquatic sources, and the first isolation and characterization of an *M. ulcerans* strain from an aquatic Hemiptera (water striders, *Gerris* sp.) from Benin was reported by Portaels *et al.* [6]. The individuals living in the same environment appear to have different susceptibilities to the disease. Indeed, most individuals exposed to *M. ulcerans* never develop BU disease [7].The reason why some individuals, but not others, exposed to *M. ulcerans* develop BU is unknown but it could be linked to individual differences in innate and acquired immune responses to infection by this bacterium. Furthermore, the susceptibility to the development of BU may be determined by genetic factors as well. Several studies have been conducted to determine the genetic and/or immunological factors affecting BU disease [8]–[10], but no studies have examined family relationship as a factor of presumptive susceptibility to BU. The aim of our study was to determine whether the occurrence of BU is associated with family relationship or the practice of consanguineous marriage, in addition to daily contact with natural water sources.

## Materials and Methods

### Study design and participants

A case control study was carried out during the period of January 1^st^ to June 30^th^, 2008.

The patients included in this study as **cases** were diagnosed and followed up at the BU detection and treatment center (CDTUB) of Allada (Department of the Atlantique, Benin) or at various health care centers (HCC) involved in the treatment of BU under the supervision of the CDTUB of Allada. From January 2006 to June 2008, the cases with active BU lesions (nodule, edema, plaque, ulcer or osteomyelitis) were recruited [11] and confirmed by at least one laboratory test (direct smear examination showing acid-fast bacilli, positive culture or IS*2404*-PCR) [12]. The individuals who were no longer hospitalized were located by using the addresses in their medical file. Eligible cases who had moved or were not found during the data collection were excluded. All eligible cases who were not living in the Atlantique department were also excluded.

An eligible **control** was defined as a person who had no signs or symptoms of active or inactive BU. The eligible controls who were suffering or had suffered from any mycobacterial disease (leprosy, TB or BU) were excluded as well. Three controls, matched by age, gender and village of residence, were selected for each case. The controls were randomly sampled from within the village of the case according to the matching criteria. A door-to-door systematic procedure was used for control selection from the center of the village of each related case.

### Sample size

We used power calculation tools to determine the sample size. We set alpha equal to 0.05 and power equal to 80%. We assumed a rate of three controls per case. Because we lacked data on the frequency of consanguineous marriages, we assumed a rate of 50% in controls. The minimum of the odds ratio (OR) for the association between cases and controls was set equal to two. We obtained a sample size of 396 participants, including 99 cases and 297 controls.

### Data collection

A standard questionnaire was administered to eligible cases and matched controls (or their guardians) by trained investigators. Structured interviews were conducted with the participants during home visits using the pre-tested questionnaire translated in Aïzo and Fon (the most commonly spoken languages in the region). Interviews with current in-patients were conducted in the hospital. The questionnaire was filled out by the interviewer. If required, subsequent interviews were conducted until all the required data were obtained.

The participant's identification data (age, gender, geographical origin), family history regarding any disease (especially sickle cell disease, diabetes and arterial hypertension), marital status (single or not) and habits regarding daily contact (contacts from professional or domestic activities and, in the case of children, from play activities) with natural sources of water (e.g., river, lake, lagoon, swamp) were collected. The data relating to the illness (clinical form, site and categorization of the lesion based on the World Health Organization (WHO) definition [11]) were also collected.

The family history of BU in the participant was investigated and if present, the number of family members who had BU was recorded. For each family member who had BU, the data were collected on the degree of the relationship with the participant (grandparents, parents, collaterals and descendants), the residence at the time he/she was ill (same house, same village but not the same house or other village or town) and whether or not he/she had daily contact with a natural water source during his/her daily activities.

The data were collected on the existence or practice of consanguineous marriages. When found, the type of relationship between the married couple (brother/sister, cousins, parents/children, uncle/niece or aunt/nephew) and the degree of the relationship in the consanguineous married couple to the participant were determined.

The pedigree of each participant was determined (with the help of the parents or guardians) using an in depth interview. The pedigrees went as far back or forward as the 3^rd^ generation before or after the participant, when applicable, and included the collaterals. Every parent who had BU was carefully specified.

### Statistical and data analyses

The data were recorded and analyzed using epiinfo 3.5.1 (Database and statistics software for public health professionals, Centers for Disease Control and Prevention (CDC), Atlanta, USA).

First, a descriptive univariate analysis was conducted on the characteristics of the participants, using Pearson's chi-square test or Fisher test. Second, the cases and the controls were compared using both univariate and multivariate analysis to determine the odds ratio (OR) and its 95% confidence interval (95% CI). To examine the association between family relationships and the occurrence of BU, all variables were included in a multiple conditional logistic regression model, followed by a step by step backward elimination based on the likelihood ratio in which only significant predictors were retained”. The participant status (case or control) was the dependent variable; all other variables were used as independent variables. Non Respondents have been excluded from the multiple conditional logistic regression analysis.

### Ethical provisions

The study enrollment was voluntary. A written informed consent was obtained from the cases and the controls or from their parents or guardians (for patients younger than 15 years). All BU cases had received or were currently receiving free treatment for BU according to the WHO's recommended protocol [11]. The study protocol was authorized by the Ministry of Health of Benin.

## Results

### Participant characteristics

There were 416 participants in the study, including 104 cases and 312 controls.

Among the cases, the median age was 12 years (2 to 68 years). A total of 62 patients (59.6%) were <15 years and 58.7% (61 out of 104) were male. A total of 75 (72.1%) patients came from the Zê district, while 10 (9.6%) came from Allada, 9 (8.7%) from Toffo, 6 (5.8%) from So-Ava, 3 (2.9%) from Abomey-Calavi and 1 (1.0%) from Tori-Bossito. The age, gender and geographical origin were similar for the cases and the controls because of the matching protocol used in control selection. There were no statistical differences between the cases and the controls with regard to marital status, hereditary disease history and daily contact with natural sources of water (Table 1).

**Table 1:** Characteristics of BU cases and controls.

Variables	Cases [N (%)]	Controls [N (%)]	p value
Age			0.77
<15 years	62 (59.6)	191 (61.2)	
≥15 years	42 (40.4)	121 (38.8)	
Gender			
Male	61 (58.7)	183 (58.7)	
Female	43 (41.3)	129 (41.3)	
Geographical origin			
Ze	75 (72.1)	225 (72.1)	
Allada	10 (9.6)	30 (9.6)	
Toffo	9 (8.7)	27 (8.7)	
So Ava	6 (5.8)	18 (5.8)	
Abomey Calavi	3 (2.9)	9 (2.9)	
Tori bossito	1 (1.0)	3 (1.0)	
Marital status			0.38
Single	77 (74.0)	244 (78.2)	
Other	27 (26.0)	68 (21.8)	
Hereditary disease in the family			0.43
Yes	16 (15.4)	39 (12.5)	
No	88 (84.6)	273 (87.5)	
Daily contact with natural water			0.08
Yes	76 (73.1)	199 (63.8)	
No	28 (26.9)	113 (36.2)	

(Total cases/Total controls = 104/312).

With respect to clinical form, 56 cases (53.8%) had ulcerative lesions and 48 cases (46.2%) had non-ulcerative lesions. Lesions were on the lower limbs for 55 cases (52.9%), the upper limbs for 34 (32.7%) and on the trunk for 10 (9.6%). There were four cases with lesions on multiple sites (3.8%) and one case (1.0%) with a lesion on the face. The categorization of patient lesions based on the WHO definition [10] placed 10 (9.6%) in category 1, 59 (56.7%) in category 2 and 35 (33.7%) in category 3 (data not shown).

### Association between family relationships and the occurrence of Buruli ulcer: univariate and multivariate analyses

Table 2 shows the association between family relationships and the occurrence of BU based on the univariate and multivariate analyses. The univariate analysis showed that BU history in the family and daily contact with natural water source were strongly associated with an increased risk of BU (OR; 95% CI = 5.07; 2.81–9.14 and 2.31; 1.18–4.53 respectively). A consanguineous marriage in the family was not associated with the occurrence of BU (p = 0.33). In the multivariate conditional logistic regression model including participant characteristics, two main factors were retained and associated with the occurrence of BU: (1) daily contact with natural source of water (OR; 95% CI = 2.7; 1.3–5.5); (2) BU history in the family (OR; 95% CI = 5.5; 3.0–10.0).

**Table 2:** Univariate and multivariate analysis of the association between family relationships, characteristics of cases and controls and the occurrence of BU.

	Univariate analysis		Multivariate analysis	
Variables	OR; 95% CI	P value	OR; 95% CI	P value
Age <15 year-old	0.73; 0.14–3.74	0.71		
Marital status = single	0.43; 0.13–1.40	0.16		
Hereditary disease in the family	1.30; 0.67–2.52	0.43		
**Daily contact with natural water**	**2.31; 1.18–4.53**	**0.01**	**2.7; 1.3–5.5**	**0.007**
**BU history in the family**	**5.07; 2.81–9.14**	**<0.001**	**5.5; 3.0–10.0**	**<0.001**
Practice of consanguineous marriage in the family	1.49; 0.66–3.38	0.33		

OR = Odds Ratio.

(Total cases/Total controls = 104/305).

Table 3 shows the degree of the relationship between the cases and any other family member with BU. Associations between family relationships (parents, siblings, cousins, sons, daughters, nieces and nephews) and the occurrence of BU were not found. But, the BU cases were more likely to have grandparents with BU than the controls (p = 0.06). However, there was a lack of precision, the 95% CI being too large (95% CI = 0.85–64.08).

**Table 3:** Degree of relationship between the family member who had BU and the study subjects.

Variables	Univariate analysis	
(BU history in)	OR; 95% CI	P value
Grandparents	7.36; 0.85–64.08	0.06
Parents (father/mother or uncle/aunt)	1.05; 0.37–2.95	0.93
Collateral (brother/sister or cousin)	1.08; 0.42–2.80	0.87
Progeny (son/daughter or nephew/niece)	0.46; 0.05–4.51	0.50

OR = Odds Ratio.

(Total cases/Total controls = 48/60).

Seven grandparents had a history of BU, six from cases and one from a control. The grandfather was involved three times and the grandmother four times. From seven affected grandparents, two were currently living in the same house, two were living in the same village (but not in the same house), two were living outside the village (including the grandparent of the control) and one was deceased. At the time of the disease, three of the involved grandparents were living in the same house as the patient, two in the same village and one outside the village. The place of residence at the time of the disease was not known for one grandparent. There was no statistical difference with regard to the living places of the grandparents involved between cases and controls (p = 0.30, Fisher test). All grandparents related to a BU case had contact with a natural source of water during their daily activities (data not shown).

## Discussion

The objective of this study was to investigate whether or not daily contact with natural sources of water combined with family relationships was associated with an increased susceptibility to the development of BU. Our major finding was that the odds ratio of having BU was three times higher in the cases than the controls for those having a daily contact with natural sources of water, and five times higher for those who had a history of BU in their family.

Many publications have reported a relationship between BU and neighborhoods in humid environments (reviewed in [13]–[15]). Lunn *et al.* in 1965 [16] and also Barker in 1972 [17] described cases that were primarily from the Nile Valley and bordering marshes in Uganda. Ravisse in 1975 described cases in Cameroon originating from the Nyong River and surrounding swamps [18]. In 1976, Oluwasanmi described cases in Nigeria that were located in an area near an artificial lake bordering the University of Ibadan [19]. In 2005, Johnson *et al.* showed that there was an inverse relationship between the prevalence of BU and the distance from the Couffo River in Benin [20]. In 2008, Kibadi *et al.* reported three patients originating from villages near the Cuango and Kwango River in Angola and the Democratic Republic of Congo, respectively [21]. Thus, it is clear from these studies that *M. ulcerans* disease occurs mainly in areas located near rivers, lakes or swamps.

Nontuberculous mycobacteria (NTM) can be found everywhere in nature and at all latitudes. In BU endemic areas of Benin, *M. ulcerans* DNA has been detected in several aquatic organisms [22]–[24]. Humans and animals are regularly in contact with the environmental mycobacteria. Consequently, the colonization of healthy individuals by NTM is fairly common [7]. It is thus important to know why some individuals develop the disease while others do not.

Our results have shown that daily contact with a natural water source is a risk factor for BU, which confirms previous studies that investigated behavioral factors associated with BU [25]–[30].

In addition, we have shown, for the first time, the existence of family associations with BU. Previous studies have incorporated BU history in the family among the factors tested but did not find a statistically significant association [25], [29], [30]. In contrast to the other studies, we included several generations (e.g., grandparents, parents, children) in our study, which might explain the discrepancies. The observed association of BU history in the family and the occurrence of the disease provide new evidence with regard to the susceptibility to BU. This association may be due to the fact that members of the same family have perhaps same habits and same exposure to common environmental sources. However, we did not find any association between family members of the contemporaneous generations (collaterals, parents or children). This leads us to suggest that genetic factors may increase susceptibility to the BU disease.

Several studies have shown that susceptibility to other mycobacterial infections, such as TB and leprosy, involve a major genetic component that determines the susceptibility of animals and humans to these infections [31]–[38]. In these studies, the development of TB or leprosy upon exposure to the mycobacteria and the pattern of clinical manifestations displayed by patients (pulmonary TB, paucibacillary or multibacillary leprosy) were highly dependent on human genes [32]–[35], [37], [38]. Genetic susceptibility to the development of BU was demonstrated by Stienstra *et al.* [39], [40]. A similar pattern had previously been found in Leprosy and TB [32], [41]. Awomoyi *et al.* showed that *SLC11A1* (*NRAMP1*) influenced TB susceptibility by regulating immunosuppressive cytokines such as interleukine-10 (IL-10) [41], subsequently reducing the Th-1 immune response in the active disease [10]. Several studies have also demonstrated the genetic origin of cytokine deficiencies observed in mycobacterial infections [42], [43]. In addition, it is known that subjects with past or current *M. ulcerans* infection mount a dominant Th-2 type response (as also observed in advanced TB [10], [44], [45]) following stimulation with *M. ulcerans*, while unaffected contacts responded mainly with a Th-1 type response [10]. Thus, the various clinical lesions (nodule, plaque or edema), as well as the resistance to BU, may rely on host factors, such as the type of immune response, that depend on genetic factors. This suggests that patients who develop the clinical disease and those who develop a severe form of the disease appear to have an inherent inability to generate a strong Th-1 response to mycobacterial antigens [10]. Characterizing these primary immunodeficiencies helped to define the Mendelian susceptibility to mycobacterial infections syndrome (MSMIS) [36], [42], [46], [47]. Further studies should explore which human genetic factors play a role in BU infection per se, and in the development of its different clinical forms. This pattern may be of great therapeutic importance.

A gene is inherited in a dominant, recessive or co-dominant mode. Depending on the mode of transmission, the susceptibility to infection can be maintained from one generation to the next in a continuous, discontinuous or even random way. In our study there could be an association between the existence of BU in grandparents and the occurrence of the disease in the study cases (since the p value was at the level of significance (p = 0.06)), whereas there was no association for parents, collaterals or progeny. However, our study lacks precision (95% CI = 0.85–64.08).

Consanguineous marriage is a practice that could promote an imbalance in the transmission of certain genes and thus the development of anomalies. Asha Bai *et al.* showed that developmental anomalies were significantly more frequent (p<0.001) among the progenies of consanguineous parents [48]. La Rosa, in 2008 [49], proposed the hypothesis that ethnic endogamy could explain the focal distribution of BU as described in Benin [20]. Lyons *et al.* showed that consanguinity was an important risk factor in susceptibility to infectious diseases in humans [50]. In particular, they found that cases of TB and hepatitis were more common among inbred individuals, but only in populations where consanguineous marriages are common [50]. Our study does not show any statistical difference in the frequency of consanguineous marriages between the cases and the controls. The overall frequency of the consanguineous marriage practice in our study was only 10.3%. In the cohort of Asha Bai *et al.* in India, it was 41.4% [48]. However, there is no information on the overall incidence of consanguineous marriages in Benin, sub-Saharan Africa or our study area. Thus, this is an area where further research is needed.

## Conclusion

This study confirmed the role of water contact as a risk factor and also suggests that the combination of genetic factors may constitute risk factors for the development of BU. Further studies should explore which human genetic or epigenetic factors play a role in BU infection and the development of the disease.

## Supporting Information

Checklist S1 STROBE checklist
(0.09 MB DOC)
